# Light‐Fueled Hydrogel Actuators with Controlled Deformation and Photocatalytic Activity

**DOI:** 10.1002/advs.202204730

**Published:** 2022-10-17

**Authors:** Pengyu Chen, Qiushi Ruan, Rasool Nasseri, Hanning Zhang, Xufeng Xi, Huan Xia, Gang Xu, Qian Xie, Chengjie Yi, ZhengMing Sun, Hamed Shahsavan, Wei Zhang

**Affiliations:** ^1^ Jiangsu Key Laboratory of Advanced Metallic Materials School of Materials Science and Engineering Southeast University Nanjing 211189 P. R. China; ^2^ Department of Chemical Engineering and Waterloo Institute for Nanotechnology University of Waterloo Waterloo Ontario N2L 3G1 Canada

**Keywords:** anisotropic hydrogel actuators, gold‐decorated carbon nitride, nanoparticles, photocatalysis, photoresponsive, soft robots

## Abstract

Hydrogel actuators have shown great promise in underwater robotic applications as they can generate controllable shape transformations upon stimulation due to their ability to absorb and release water reversibly. Herein, a photoresponsive anisotropic hydrogel actuator is developed from poly(N‐isopropylacrylamide) (PNIPAM) and gold‐decorated carbon nitride (Au/g‐C_3_N_4_) nanoparticles. Carbon nitride nanoparticles endow hydrogel actuators with photocatalytic properties, while their reorientation and mobility driven by the electrical field provide anisotropic properties to the surrounding network. A variety of light‐fueled soft robotic functionalities including controllable and programmable shape‐change, gripping, and locomotion is elicited. A responsive flower‐like photocatalytic reactor is also fabricated, for water splitting, which maximizes its energy‐harvesting efficiency, that is, hydrogen generation rate of 1061.82 µmol g^−1^ h^−1^, and the apparent quantum yield of 8.55% at 400 nm, by facing its light‐receiving area adaptively towards the light. The synergy between photoactive and photocatalytic properties of this hydrogel portrays a new perspective for the design of underwater robotic and photocatalytic devices.

## Graphical abstract

Hydrogel actuators with anisotropic microstructure, photothermal shape‐change, and photocatalytic activity are prepared from composites of poly(N‐isopropylacrylamide) and Au/g‐C_3_N_4_ nanoparticles. A variety of light‐fueled controllable shape‐change is elicited, applicable to soft robotics. The photothermal deformation of hydrogel constructs is utilized to maximize their energy harvesting capability and photocatalytic hydrogen generation.

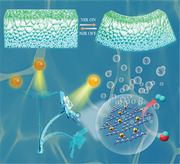

## 1 Introduction

Bio‐inspired soft actuators that convert the energy received from external stimuli, such as heat or light, into mechanical work through flexible and complex shape‐change and motion have been the subject of extensive studies in the past decade.^[^ ^1^, ^2^, ^3^, ^4^, ^5^, ^6^ ^]^ Biomimetic shape‐changes of soft actuators, which can be harvested for locomotion, such as swimming, jumping, and crawling, or function, such as gripping and cargo transport, have received growing interest in soft robotics, tissue engineering, and medical devices.^[^ ^7^, ^8^, ^9^ ^]^ However, many soft actuators, including liquid crystal elastomers, shape memory polymers, and electroactive polymers, have a restricted degree of freedom in actuation, mobility, biocompatibility, and functionality in aqueous environments due to the technical sophistication and large energy requirements of underwater stimulation,^[^ ^10^, ^11^, ^12^ ^]^ which limits their performance in many biological and marine‐related applications. As alternatives, stimuli‐responsive hydrogels offer desirable actuation advantages in complex underwater conditions, such as lower energy requirement for actuation, adjustable response time, biocompatibility, and tunable modulus equivalent to biological tissue.^[^ ^13^, ^14^ ^]^ Most of the current hydrogel actuators are crosslinked hydrophilic networks with a high water content that can produce controllable shape transformations upon stimulation due to their ability to absorb and release water reversibly.

Among common external stimulation sources, such as light,^[^ ^15^, ^16^ ^]^ electricity,^[^ ^17^, ^18^ ^]^ magnetic field,^[^ ^19^, ^20^, ^21^ ^]^ heat,^[^ ^13^, ^22^, ^23^ ^]^ pH,^[^ ^24^, ^25^ ^]^ and chemicals,^[^ ^2^ ^]^ near‐infrared (NIR) light has shown great potential in remote stimulation of photoresponsive materials with high selectivity, and superior tissue penetration.^[^ ^26^, ^27^ ^]^ Stimulation of photoresponsive hydrogel actuators with NIR, indeed, allows for precise spatiotemporal control of their shape‐change without damage or contact underwater.^[^ ^28^ ^]^ Poly(N‐isopropylacrylamide) (PNIPAM) hydrogel is a classic example that exhibits a distinct thermo‐responsive solubility behavior near its lower critical solution temperature (LCST). This feature has been utilized for macroscopic shape‐change including expansion/shrinkage, bending, or other complex modes of deformation. PNIPAM‐based hydrogels doped with light‐absorbing materials that can convert NIR to macroscopic shape‐change and locomotion have been widely used to prepare photo‐responsive hydrogel actuators.^[^ ^29^, ^30^ ^]^

The presence of an anisotropic microstructure is key for programming the shape‐change of hydrogels. Several anisotropic configurations including patterned structure,^[^ ^31^, ^32^ ^]^ double‐layer structure,^[^ ^33^, ^34^, ^35^ ^]^ Janus structure,^[^ ^36^ ^]^ and gradient pore structure^[^ ^22^, ^37^ ^]^ were developed to fabricate well‐defined hierarchical anisotropic microstructures. Among them, modulation of pore size and pore size distribution to endow PNIPAM hydrogels with gradient microstructure has been demonstrated as an effective approach to tailor their mechanical strength, response time, shape‐change, and actuation behaviors.^[^ ^38^ ^]^ Inducing heterogeneous distributions of anisotropic filler particles during the polymerization has been used for the fabrication of various anisotropic PNIPAM hydrogels with cross‐linking gradience with respect to the direction of gravity, electric, or magnetic field.^[^ ^39^ ^]^ The inclusion of such filler particles can also enhance hydrogels’ response time, reversibility, reconfiguration, and control over movement upon NIR irradiation.^[^ ^40^ ^]^ Reduced graphene‐oxide (rGO), montmorillonite, MXene, and anisotropic magnetic nanoparticles are some common examples of functional filler particles.^[^ ^22^, ^38^, ^40^, ^41^, ^42^ ^]^ These functional fillers are often applied to induce anisotropy inside hydrogels and to elicit another function, such as magnetic response, or photothermal heating. Other potential synergetic functions arising from their intrinsic optical, electrical, or catalytic properties are seldom utilized. Recently hydrogen generation through water splitting using photothermal‐photocatalytic particulate‐based systems, such as systems containing perovskites, MXenes, and others, is deemed one of the most economical, efficient, and eco‐friendly approaches. In the majority of published work, steam generated by the photothermal functionality of the system is split via photocatalytic functionality in a static manner.^[^ ^43^, ^44^, ^45^, ^46^, ^47^, ^48^, ^49^ ^]^

**Figure 1:**
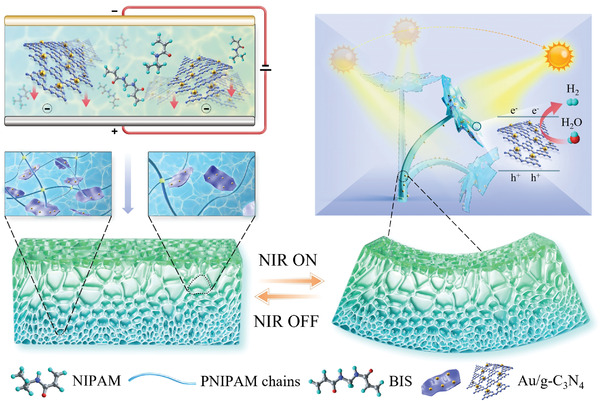
Schematic illustration of Au/g‐C_3_N_4_‐containing gradient hydrogel actuator preparation, shape transformation, and photocatalytic phototropic behavior.

Herein, we report a multisectional hydrogel construct that employs the photothermal dynamic shape‐change to maximize the exposure of its photocatalytic reactor portion to the incident light to enhance the water‐splitting performance. For this, we have developed a photoresponsive anisotropic hydrogel actuator based on PNIPAM and gold‐decorated graphite phase carbon nitride (g‐C_3_N_4_) nanoparticles (NPs). g‐C_3_N_4_ NPs endow PNIPAM hydrogels with anisotropic as well as photocatalytic properties, which can be efficiently harvested for water splitting coupled with light‐fueled deformation (**Figure** **1**). g‐C_3_N_4_ possesses a broad absorption spectrum, suitable bandgap, excellent chemical stability, high thermal conductivity, and tunable structure and properties.^[^ ^50^, ^51^ ^]^ When mixed with the hydrogel matrix, g‐C_3_N_4_ can induce gradient distribution of the polymer chains subject to an external electric field. g‐C_3_N_4_ also provides the hydrogel with photocatalytic properties. Its band edge position satisfies the redox potential required for water splitting. The absorbed energy of photons generates electrons and holes that migrate to the surface of g‐C_3_N_4_ for subsequent redox reactions (Figure S1, Supporting Information).^[^ ^52^, ^53^ ^]^ Using such properties of g‐C_3_N_4_, we induced a structural anisotropy to form a gradient hydrogel network. Then we demonstrated various controllable and programmable shape‐change and motions of the hydrogel actuator under NIR light stimulation. Finally, we prepared a bionic flower‐like photocatalytic “hydrogel reactor”, which can maximize its energy‐harvesting and photogenerated hydrogen production via water splitting by facing its light‐receiving area toward external illumination (Figure 1). The H_2_ generation rate of the hydrogel photocatalytic device under two opposite illumination directions was determined to be 950.9 and 1061.82 µmol g^−1^ h^−1^ and the apparent quantum yield (AQY) reached 7.29% and 8.55% at 400 nm, which demonstrated superior photocatalytic performance among other g‐C_3_N_4_‐based hydrogels in literature (Table S1, Supporting Information). These results revealed that the functional fillers can not only be used to construct anisotropic hydrogel actuators with programmable photothermal properties but also endow them with new functionalities, demonstrating promising prospects in biological and marine‐related applications.

## 2 Results and Discussion

**Figure 2:**
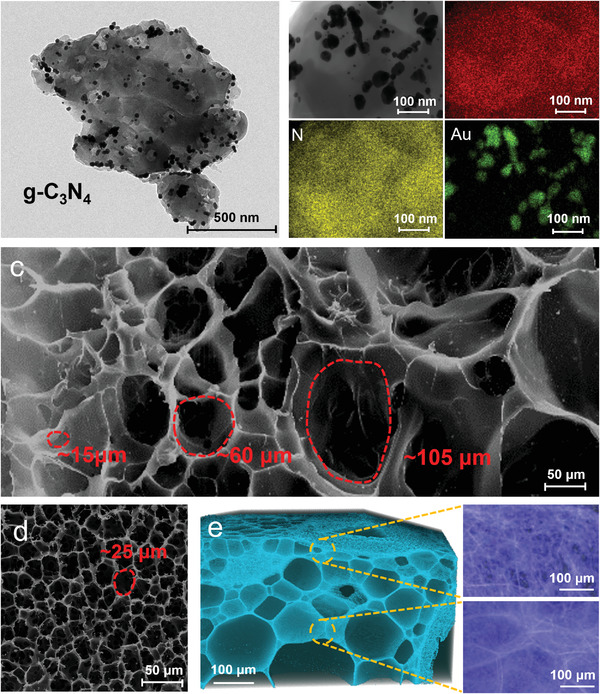
a) A TEM micrograph of Au/g‐C_3_N_4_ particles; b) The elemental‐mapping images of Au/g‐C_3_N_4_ nanoparticles showing C, N, and Au; c) An SEM micrograph of the E_2.5_C_10_ gradient hydrogel sample from its cross‐section; d) An SEM micrograph of the E_0_C_10_ homogeneous hydrogel sample from its cross‐section; e) Nano‐CT images of the E_2.5_C_10_ sample and local magnified images.

To maximize the photothermal properties of the PNIPAM hydrogels, gold nanoparticles (AuNPs) were attached to the surface of g‐C_3_N_4_ by reduction of chloroauric acid with sodium citrate to form Au/g‐C_3_N_4_ nanocomposites. The high‐resolution transmission electron microscopy (HRTEM) (**Figure** **2**a) and the elemental mapping of energy dispersive spectrometry (Figure 2b) images confirm that AuNPs are uniformly deposited on the surface of g‐C_3_N_4_. The interplanar spacing of the AuNPs is approximately 0.23 nm, which is consistent with the XRD patterns of the Au (111) plane (Figures S2 and S3, Supporting Information). The ultraviolet‐visible (UV–vis) analysis results prove that the Au/g‐C_3_N_4_ composites exhibit higher optical absorption in the visible and infrared range (300–900 nm) than pristine g‐C_3_N_4_ (Figure S4, Supporting Information), and the optical absorption of the composites becomes more pronounced as the Au concentration increases. Such increased absorption can be attributed to the strong absorption of photon energy by AuNPs which undergo localized surface plasmon resonance (LSPR).^[^ ^54^ ^]^

The aqueous solution of g‐C_3_N_4_ NPs exhibits a large negative Zeta potential due to the large electronegativity of the accumulated amino groups on its surface. (Figure S5, Supporting Information). Decoration of g‐C_3_N_4_ with gold does not affect the Zeta potential significantly, which is essential for their ability to migrate in the presence of electric fields. The characteristic absorption peak of the surface amino group at 3160 cm^−1^ was observed for pure g‐C_3_N_4_ in the Fourier transform infrared spectroscopy^[^ ^55^ ^]^ (Figure S6, Supporting Information). After incorporation of NPs into the hydrogel matrix, the absorption peak of ‐C=O stretching was shifted from 1645 cm^−1^, for the pristine PNIPAM hydrogel, to 1640 cm^−1^, for the Au/g‐C_3_N_4_‐PNIPAM hydrogel. This small shift is probably due to the formation of hydrogen bonds between the amino groups on the surface of the g‐C_3_N_4_ and oxygen from the polymer chains.

To induce anisotropic microstructure to the hydrogel, a DC electric field was applied during the preparation, which caused the migration of the negatively charged Au/g‐C_3_N_4_ NPs towards the anode. Such anisotropic distribution of NPs simultaneously led to a concentration gradient of precursor monomers or short chains along the direction of the electric field. The gradual distribution of the PNIPAM chains was achieved by in situ free‐radical polymerization and chemical cross‐linking with N, N′‐methylene bis(acrylamide) (BIS), leading to a gradient porous network. For brevity, the hydrogel samples were named E_a_C_b_, where a and b represent the intensity of the electric field (V mm^−1^) and the mass concentration of Au/g‐C_3_N_4_ (mg mL^−1^), respectively. Scanning electron microscopy (SEM) images of the E_2.5_C_10_ hydrogel sample (Figure 2c) show the pore size distribution along the electric field thickness direction. The hydrogel network near the anode side is much denser and has a smaller average pore size of 15 µm, while the network around the cathode side is much looser with a larger average pore size of approximately 105 µm. In contrast, the hydrogel samples prepared in the absence of an external electric field show a relatively homogeneous network structure (Figure 2d). We also used Nano‐Computed Tomography (NCT) to gain more insight into the network microstructure of hydrogel samples. 3D reconstructed images of the E_2.5_C_10_ hydrogel (Figure 2e) provide a clearer visualization of the hydrogel microstructure and vividly illustrate the asymmetric distribution of the porous network. The difference in pore size can also be observed from the local magnified images. These results demonstrate that the formation of the gradient network is related to the distribution of g‐C_3_N_4_ under the action of an electric field.

**Figure 3:**
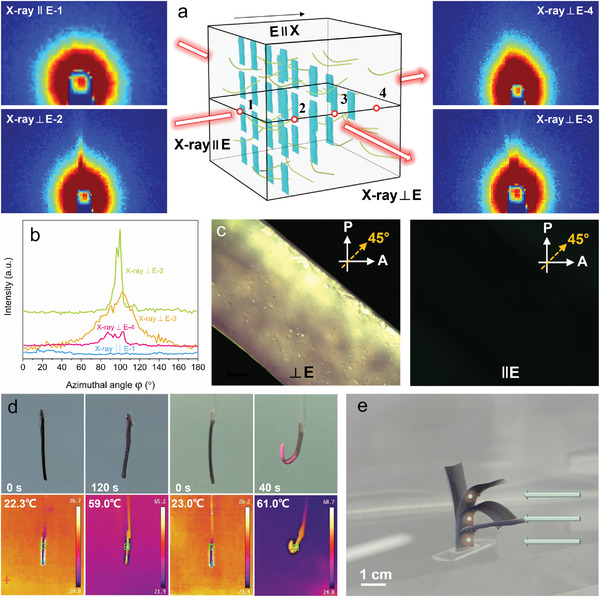
a) SAXS images of Au/g‐C_3_N_4_‐containing gradient hydrogel measured from parallel directions to the applied electric field (X‐ray∥E‐1); and the ones measured from perpendicular direction to the applied electric field, X‐ray⊥E‐2, X‐ray⊥E‐3, and X‐ray⊥E‐4 correspond to different positions; b) Azimuthal angle (*φ*) plots for the SAXS images; c) POM images of the E_2.5_C_10_ hydrogel sample observed from different directions (⊥E and ∥E); d) Real and infrared images of the E_0_C_10_ hydrogel and the E_2.5_C_10_ hydrogel actuator in the air upon exposure to 808 nm NIR light (0.5 W cm^−2^); e) Bending superimposed snapshots of the E_2.5_C_10_ hydrogel long strip at different light positions.

The microscopic alignment of Au/g‐C_3_N_4_ was investigated by using small‐angle X‐ray scattering analysis (SAXS), which can shed light on the anisotropic properties of the g‐C_3_N_4_ PNIPAM hydrogels. As shown in **Figure** **3**a, the E_2.5_C_10_ gradient hydrogel exhibits elliptical diffuse patterns when the direction of X‐rays is orthogonal to the applied electric field during the preparation (⊥E), while illumination parallel to it (∥E) exhibits an isotropic pattern. Accordingly, the azimuthal angle (*φ*) plot of the SAXS pattern measured from the perpendicular direction to the electric field shows a peak at 90°, whereas the measurement parallel to the electric field is almost flat. Using the obtained azimuthal angle plots, the orientation degree of Au/g‐C_3_N_4_ NPs in the hydrogel network can be calculated by the following equation (Equation (1)):^[^ ^56^ ^]^

(1) $$\Pi =\left(180^{\circ }-H^{\circ }\right)/180^{\circ }$$

where Π is the degree of orientation and *H* represents the full width at half maximum of the I‐azimuthal angle curve. The alignment of Au/g‐C_3_N_4_ in the hydrogel is relatively uniform, with an order parameter of 0.86 (point 2, Figure 3b) at an applied voltage of 2.5 V. It is noteworthy that measurements at different positions (point 2 is closer to the anode, while point 4 is closer to the cathode) perpendicular to the electric field exhibit different intensities (Figure 3b). These results show that Au/g‐C_3_N_4_ NPs not only move toward the anode and create a gradient in porosity and NP concentration but also reorient themselves and create local alignment due to the electrostatic repulsion between NPs. A polarizing optical microscope (POM) was also used to visualize the anisotropic distribution of Au/g‐C_3_N_4_ nanosheets inside the E_2.5_C_10_ hydrogel parallel and perpendicular to the direction of the applied electric field. The sample was positioned diagonally (with a 45°) between crossed polarizers. The left panel of Figure 3c shows the cross‐section of the hydrogel sample, where a strong and uniform birefringence is observed along the thickness, equivalent to ⊥E in Figure 3a. The right panel of Figure 3c shows the top/bottom of the sample dark and with no birefringence in the planes parallel to the electrodes, equivalent to ∥E in Figure 3a. A 45° rotation of the sample causes complete extinction of light on the left panel and no difference on the right panel. These results further support that the nanosheets are aligned in the direction perpendicular to the electric field similar to some previous reports.^[^ ^35^ ^]^

PNIPAM hydrogels have a LCST of about 32 °C, and when the temperature is above the LCST, water is expelled from the polymer network, leading to structural shrinkage. Absorption of NIR light by Au/g‐C_3_N_4_ NPs in the hydrogel facilitates photothermal heating and accordingly shrinkage of the network. The shrinkage for samples prepared in the presence of an electric field is expected to be asymmetric due to the gradient of network formation and porosity. The hydrogel contracts more on the side closer to the anode and less on the side closer to the cathode, resulting in bending deformation (Figure S7, Supporting Information). To demonstrate the effect of AuNPs in enhancing the response to NIR, we compared the bending deformation of the E_2.5_C_10_ hydrogel and the control sample without AuNPs in the air (Figures S8 and S9, Supporting Information). Infrared imaging of the E_2.5_C_10_ hydrogel exposed to NIR shows an increase in the temperature from 23 to 61 °C resulting in a large bending deformation in 40 s. On the other hand, the control sample hydrogel does not show any deformation within 120 s of exposure to NIR, and its temperature remained essentially the same. The effect of the electric field during the polymerization was investigated by comparing the photothermal deformation of E_0_C_10_ and E_2.5_C_10_ samples. Unlike bending deformation of E_2.5_C_10_ upon exposure to NIR, E_0_C_10_ hydrogel undergoes isotropic contraction (Figure 3d). Such a meaningful difference in the deformation indicates that the electric field‐induced gradient network is essential for the actuation performance. The bending deformation of the E_2.5_C_10_ hydrogel actuators can be precisely controlled by adjusting the location and angle of the incoming light beam (Figure 3e).

**Figure 4:**
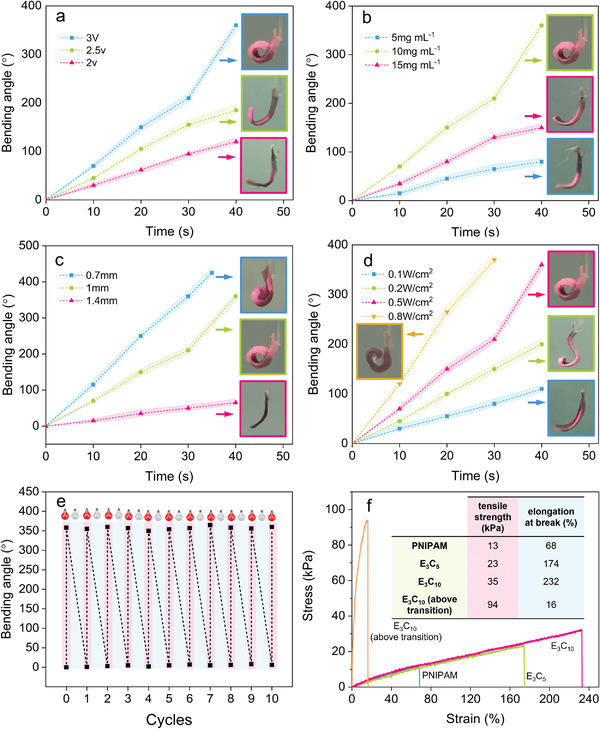
a) Bending performance for gradient hydrogel actuators (E_a_C_10_) fabricated with different applied DC electric fields upon 808 nm NIR irradiation (0.5 W cm^−2^), a = 2, 2.5, and 3; b) Bending performance for gradient hydrogel actuators (E_3_C_b_) fabricated with different concentration of Au/g‐C_3_N_4_ upon 808 nm NIR irradiation (0.5 W cm^−2^), b = 5, 10, and 20; c) Bending performance for gradient hydrogel actuators (E_3_C_10_) fabricated with different thickness (0.7, 1, and 1.4 mm); d) Bending performance for gradient hydrogel actuators (E_3_C_10_) fabricated with different NIR power density (0.1, 0.2, 0.5, and 0.8 W cm^−2^); e) Cycle performance for gradient hydrogel actuators (E_3_C_10_) upon 808 nm NIR irradiation (0.8 W cm^−2^); f) The hydrogel stress‐strain curves of the pure PNIPAM, the E_3_C_5_, and E_3_C_10_ before and after bulk phase transition.

Bending deformation of the soft actuators made from gradient hydrogels can be fine‐tuned by varying the applied electric field driving voltage, the composition of the precursor, actuators’ geometry, and the incident light intensity. With the same Au/g‐C_3_N_4_ concentration (10 mg mL^−1^) and NIR light intensities (808 nm, 0.5 W cm^−2^), hydrogels showed greater bending deformation and faster response as the applied voltage during the polymerization increased (**Figure** **4**a and Figure S10, Supporting Information). We postulate that the higher the voltage the more pronounced pore size disparity through the thickness of the hydrogel. The bending angle *θ* of the gradient hydrogel can also be tuned by varying the concentration of Au/g‐C_3_N_4_ NPs in hydrogel precursors. For instance, illumination of E_3_C_b_ samples (b = 5, 10, and 15 mg mL^−1^) with identical NIR light intensity (808 nm, 0.5 W cm^−2^) showed an interesting trend. As can be seen in Figure 4b and Figure S11, Supporting Information, E_3_C_10_ hydrogel has a maximal bending deformation, probably due to the trivial microstructural gradient for hydrogels with lower NP concentration. On the contrary, excessive NPs could frustrate the contraction of polymer chains during the phase transition. The deformation behavior can also be tuned by changing the thickness of the actuator and maintaining the illumination intensity, composition, and applied voltage during the polymerization. As shown in Figure 4c and Figure S12, Supporting Information, the extent and speed of actuation decrease with the increase of hydrogel thickness. We speculate that the order parameter and anisotropy along the thickness are adversely affected by increasing the thickness while maintaining the applied electric field, leading to less pronounced photothermal bending. Higher NIR light intensities result in faster response and larger bending deformation. The hydrogel actuator indeed showed a bending deformation of approximately 370° within 30 s (Figure 4d and Figure S13, Supporting Information), when illuminated by 0.8 W cm^−2^ of 808 nm NIR.

In order to use the developed soft actuators in soft robotic applications, they should maintain their mechanical integrity to provide a reasonable output work over multiple cycles of use. The amplitude of the photothermal bending of some of our hydrogel actuators, such as E_3_C_10_, remained nearly unchanged even after 10 cycles of actuation in an aqueous environment (Figure 4e). Such excellent reversibility and durability of hydrogel actuators could be attributed to the fact that Au/g‐C_3_N_4_ NPs could in fact enhance the cross‐linking and further reinforce the hydrogel network.^[^ ^57^ ^]^ To shed more light on actuators’ durability, we evaluated the mechanical properties of the hydrogels prepared under 3 V applied voltage with different Au/g‐C_3_N_4_ (0, 5, and 10 mg mL^−1^) concentrations using tensile test experiments and compared the results with that of pure PNIPAM. As shown in the stress‐strain curves (Figure 4f), the pure PNIPAM hydrogel exhibits 68% elongation at break and 13 kPa tensile strength. An increase in Au/g‐C_3_N_4_ concentration enhances the mechanical properties of hydrogels significantly. For example, the tensile strength and elongation at break increase up to 169% (35 kPa) and 241% (232%) by adding 10 mg mL^−1^ of Au/g‐C_3_N_4_. When the E_3_C_10_ hydrogel undergoes a bulk phase transition, the hydrogen bonds between the hydrophilic amide groups and water molecules are weakened, the hydrophobic interactions among the isopropyl groups become strong, and water is expelled from the hydrogel network. Coil to globule transition of the chains followed by their association into polymer‐rich domains,^[^ ^58^ ^]^ and a higher number of physical cross‐links leads to a much larger tensile strength (94 kPa) (Figure 4f).

Excellent mechanical properties and durability of synthesized hydrogel actuators enabled us to use them in several proof‐of‐concept underwater soft robotic applications, including reversible shape‐change, locomotion, and cargo transport.

**Figure 5:**
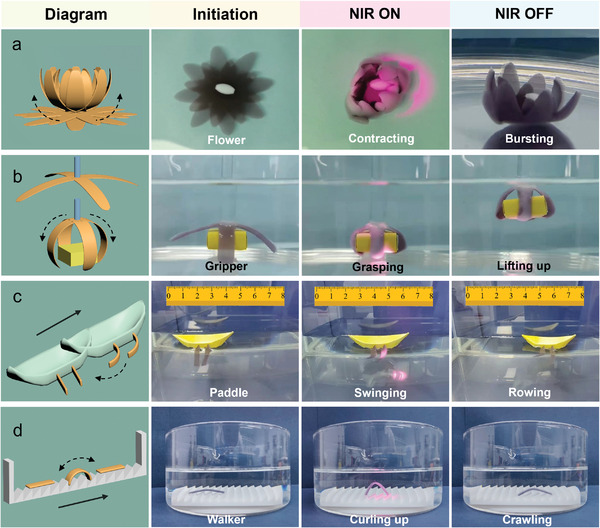
a) The opening and closing action of an end effector from composite hydrogel actuators; b) The hydrogel four‐arm gripper that can grasp, move and lift cargo underwater; c) The hydrogel paddle actuator propelling a boat‐like construct upon consecutive NIR illumination; d) The hydrogel actuator walking on a ratchet surface upon consecutive NIR illumination.

First, we fabricated a bioinspired end‐effector that mimics the opening and closing of flowers in water under NIR light irradiation (**Figure** **5**a and Movie S1, Supporting Information). Further, we used such a design to fabricate a light‐driven soft gripper. When the gripper is lit by NIR light, it bends and wraps around the cubic cargo. The excellent mechanical properties of the hydrogel allow for the lifting of the cargo (Figure 5b and Movie S2, Supporting Information), while its reversible actuation facilitates the gradual release of the cargo and retains its initial shape when the illumination stops. We also connected hydrogel actuators to a boat‐like construct. Hydrogel actuators underwent consecutive bending and relaxation by switching NIR ON and OFF and propelled the boat‐like construct forward similar to a motion driven by paddling (Figure 5c and Movie S3, Supporting Information). We believe that the motion of the boat at the water‐air interface is mainly due to such consecutive bending and relaxation of hydrogels, and not other possible mechanisms such as the thermally‐driven Marangoni effect. To rule out such a possibility, we repeated the same experiment this time with homogeneous E_0_C_10_ control hydrogel actuators, and boats connected to control actuators did not show any movements under NIR light. At last but not least, we elicited light‐fueled underwater locomotion of our hydrogel actuators by walking on a directional ratchet surface. As shown in Figure 5d and Movie S4, Supporting Information, the hydrogel walker underwent bending when the middle part was exposed to NIR light and recovered after the light was shut off. At every ON and OFF cycle, the center of the mass of the hydrogel was moved slightly forward facilitated by the anchoring of one of the actuator ends to the vertical part of the ratchet. By repeating this process, the hydrogel walker could perform movements in a single direction that could be defined by the ratchet. These results introduce Au/g‐C_3_N_4_‐containing anisotropic hydrogel actuators as a new class of soft functional actuators that could be utilized in underwater soft robotics and bionic devices.

**Figure 6:**
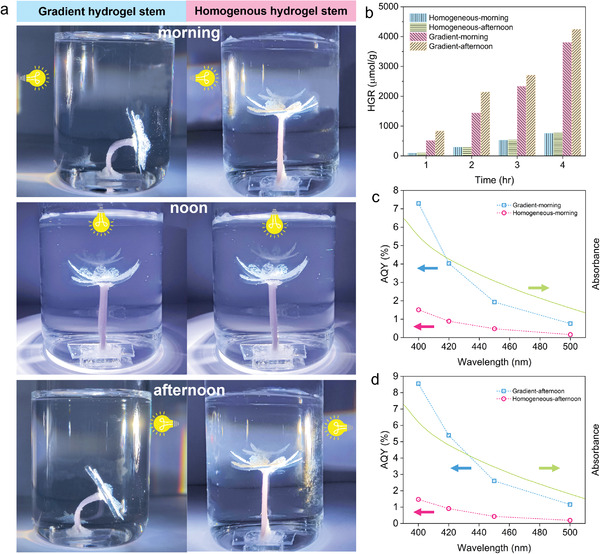
a) The bionic flower from photoactive composite hydrogel and the control passive sample is exposed to a light source from three different directions to mimic the position of the sun in the morning, noon, and afternoon; b) H_2_ generation rates of tested samples in the morning and afternoon with different exposure periods; c,d) The AQY of hydrogel flowers with active and passive stems at different wavelengths in the morning and afternoon, respectively.

In addition to inducing anisotropy, and photothermal responsiveness that Au/g‐C_3_N_4_ NPs endow to the hydrogel network, they are expected to show photocatalytic activities, which could be used for hydrogen generation via water splitting. A combination of these properties offers attractive functionalities for underwater actuators. For instance, inspired by the phototropism of sunflowers we prepared an artificial flower that can maximize its energy harvesting capability by facing its light‐receiving area towards the light source through photothermal bending. For this, Au/g‐C_3_N_4_‐PNIPAM gradient and g‐C_3_N_4_‐PNIPAM homogeneous hydrogels were fabricated to mimic the flower stem and flower petals, respectively. The petals acted as a photocatalytic reactor, and the stem was used to realize photothermal bending. As a control, an artificial flower was made using g‐C_3_N_4_‐PNIPAM homogeneous hydrogels as stems and petals. Both samples were placed in a quartz container full of an aqueous solution of triethanolamine for a half‐reaction of photocatalytic hydrogen production. We used a 150 W xenon lamp for illumination. We expected that the light in the NIR region would render the photothermal actuation and the light in the visible region would trigger photocatalytic reactions. By shining the light from three different positions, we mimicked the orientations of the sun at different times of the day (morning, noon, and afternoon). When light shines from the left side shown in **Figure** **6**a (morning), the gradient hydrogel stem bend in the opposite direction to the light source. Albeit, the light‐receiving petals of the artificial flower can still harvest a large amount of light. The sample with a passive stem does not bend and has a lower light‐harvesting capability. At noon, both samples face directly toward the light source, hence having a maximal light‐harvesting. When the light shines from the right side (afternoon), the gradient hydrogel stem orients toward the light to maintain the maximum light exposure (Figure 6a and Movie S5, Supporting Information), while the sample with a passive stem does not bend to the light‐incoming direction. The light‐harvesting ability of both samples at different illumination conditions manifests in photocatalytic activities. As can be seen in Figure 6d. the H_2_ generation rate of the artificial flower with the gradient hydrogel stem in the morning and afternoon reached 950.9 and 1061.82 µmol g^−1^ h^−1^, which was 5.1 and 5.5 times higher than that of the control sample (185.18 and 191.26 µmol g^−1^ h^−1^), respectively. At last but not least, we calculated the AQY of both artificial flowers to have a better insight into their photocatalytic activity and H_2_ evolution performance at different wavelengths. AQY of the flower with active stem was determined to be 7.29% and 8.55% in the morning and afternoon at 400 nm, while that of the control sample was only 1.51% and 1.47%. The H_2_ evolution ability decreases with increasing incident light wavelength, suggesting that it corresponds to the optical absorption of g‐C_3_N_4_. Due to the increase in wavelength, the energy of incident light decreases, and thus the number of photogenerated electrons produced by excitation decreases. The combination of light‐driven hydrogel actuators and photocatalysis through this strategy provides a new perspective for the efficient utilization of solar energy. By constructing an internal anisotropic structure, the photocatalytic efficiency of hydrogen generation is significantly improved, which could be more promising in large‐scale applications.

## 3 Conclusion

In summary, we presented a facile strategy to prepare light‐driven PNIPAM hydrogel actuators containing Au/g‐C_3_N_4_ nanoparticles. Thanks to the excellent photothermal effect of Au and the anisotropic shape of g‐C_3_N_4_ nanoparticles, we induced an anisotropic gradient microstructure along the thickness of hydrogel composites using external electric fields. Upon stimulation with NIR, obtained gradient hydrogels exhibit reversible shape‐change which can be programmed by various parameters, including the applied electric field during the polymerization, loading of nanoparticles, the geometry of actuators, and the intensity of light. The composite hydrogel actuators showed significantly enhanced mechanical properties compared to their non‐loaded counterparts as a result of the auxiliary crosslinking g‐C_3_N_4_ NPs provided in the hydrogel network. Leveraging from such properties, we used the composite hydrogel actuators in the design of soft robots with various functions, including cargo transport and locomotion. Moreover, we also demonstrated a new strategy to utilize the photocatalytic properties of g‐C_3_N_4_, simultaneously with photothermal actuation of gradient hydrogel actuators. We prepared an artificial hydrogel flower that can maximize its light harvesting in a mechanism inspired by phototropism. Note that our hydrogel system, unlike actual phototropic systems, shows only one‐sided bending upon exposure to the light due to the linear gradient morphology along its thickness. One potential solution to perfectly assimilate natural phototropism is to make a cylindrical hydrogel with radial gradient morphology to facilitate omnidirectional bending of the hydrogel towards or away from the light source. Albeit we believe that the proof‐of‐concept presented in this work provides new perspectives for the development of responsive hydrogel actuators and bioinspired soft robots and their integration into photocatalytic devices.

## 4 Experimental Section

### Synthesis of g‐C_3_N_4_

5 g carbamide (Aladdin) was placed in a crucible and calcined at 600 °C for 4 h with a temperature ramp of 5 °C min^−1^. The obtained product was then put into a pestle and mortar and ground with alcohol, and dried into g‐C_3_N_4_ nanosheets.

### Synthesis of Au/g‐C_3_N_4_ Composites

10 mg g‐C_3_N_4_ was dispersed into 30 mL deionized water and sonicated for 30 min. 100 µL of 20 wt.% Chloroauric acid (HAuCl_4_, Energy Chemical) solution was injected into the above‐mentioned solution. Then, 0.04 g sodium citrate (Aladdin) was added under vigorous stirring at 50 °C. After stirring for 6 h, the resulting solution was centrifuged for 5 mins, washed with deionized water several times, and dried in an oven at 80 °C for 10 h.

### Preparation of Au/g‐C_3_N_4_‐PNIPAM Gradient Hydrogels

The anisotropic Au/g‐C_3_N_4_‐PNIPAM hydrogels were prepared by utilizing a direct‐current (DC) electric field to induce gradient distribution of Au/g‐C_3_N_4_ composites into thermosensitive PNIPAM hydrogels. Au/g‐C_3_N_4_ (5, 10, and 15 mg mL^−1^) were first dispersed in deionized water under stirring in an ice water bath. Then, 1.05 g N‐isopropylacrylamide (NIPAM, TCI) monomer and 12 mg N, N“‐methylenebisacrylamide (BIS, Aladdin) cross‐linker were added into the solution with argon gas injected for 10 min. 20 µL 20wt.% ammonium persulfate (APS, Aladdin) solution (initiator) and 10 µL of N,N,N”,N'‐tetramethyl‐ethylenediamine (TEMED, Aladdin) (catalyst) were added into the solution. Finally, the dispersion was rapidly injected into a homemade indium tin oxide glass mold of 50 × 50 × 1 mm and kept for 10 min under DC electric field (2, 2.5, and 3 V mm^−1^). Finally, the electric field was removed, and the mold was kept for 24 h at room temperature to obtain the gradient hydrogels. In this work, the hydrogel samples were named E_a_C_b_, where a and b represent the intensity of the electric field (V mm^−1^) and the mass concentration of Au/g‐C_3_N_4_ (mg mL^−1^), respectively. The g‐C_3_N_4_‐PNIPAM hydrogels without AuNPs and the E_0_C_b_ hydrogels without applying DC electric field were prepared with the same method.

### Preparation of Bionic Sunflower

10 mg g‐C_3_N_4_ were first dispersed in 2 mL deionized water under stirring within an ice water bath. Then, 0.21 g N‐isopropylacrylamide monomer and 2.4 mg N, N'‐methylenebisacrylamide cross‐linker were added with argon gas injected for 2 min. 100 µL 0.8wt.% Chloroplatinic acid (H_2_PtCl_6_, Energy Chemical) solution (catalyst), 4 µL 20wt.% APS solution (initiator), and 2 µL of TEMED (catalyst) were added, then the solution was poured into a flower‐shaped mold. The as‐prepared gradient hydrogel was cut into long strips and placed vertically in the above containing the precursor solution flower‐shaped mold. After polymerization, the flower and the stem can be connected by forming a cross‐linked network at the interface. Finally, the whole structure was immersed in deionized water to remove the residual solution.

### Characterization

The phase ingredients of Au/g‐C_3_N_4_ composites were analyzed by a Haoyuan DX‐2700BH powder X‐ray diffractometer using Cu‐K*α* radiation (*λ* = 1.5406 Å) with MDI Jade software. SEM was performed on an FEI Sirion with 20 KV working voltage. All SEM samples were immersed in the liquid nitrogen immediately to maintain the structure of hydrogels, followed by freeze‐drying in the freeze‐dryer (SCIENTZ‐12N, China) for 12 h. Transmission electron microscopy (TEM) was performed using an FEI Tecnai G2T20 and the working voltage was set as 20 KV. The Nano‐CT images were characterized by 3D X‐ray microscopy (Zeiss Xradia 510 Versa). UV–vis‐IR transmittance was performed on a Shimadzu UV 3600 with a wavelength from 300 to 900 nm. During the test, samples were attached to a quartz glass sheet and then fixed onto the holder of the instrument. Before the test, samples were dispersed into deionized water. The Fourier transform infrared (FTIR) spectra of g‐C_3_N_4_ and Au/g‐C_3_N_4_ were examined at ambient temperature (Thermo Scientific Nicolet iS10). The range of wavelength was set from 4000 to 500 cm^−1^. Different from the tests of g‐C_3_N_4_ and Au/g‐C_3_N_4_, the Au/g‐C_3_N_4_‐PNIPAM hydrogel samples were tested with the ATR module. Small‐Angle X‐ray Scattering Measurement (SAXS) was carried out at Bruker NanoStar (Bruker, Germany) under X‐ray beam parallel and orthogonal to the applied electric field, respectively. The hydrogel sample was sliced into the size of 5 mm × 5 mm × 1 mm just before SAXS measurements, and was freeze‐dried at −40 °C for 24 h. For azimuthal angle plots, scattering in 2D SAXS images at q = 0.07–2.3 nm^−1^ was integrated for every 5° in azimuthal angle. The laser system (LWIRL808‐20W‐F, Beijing Laserwave Optoelectronics Technology Co.) was used for NIR light‐driving tests with a wavelength of 808 nm. The infrared thermal images of the gradient hydrogels were captured by an infrared (IR) thermal camera (HIKMICRO H11).

### NIR Light‐Driven Actuation of Gradient Hydrogels

The gradient hydrogels were cut into strips of 30 mm × 3 mm × 1 mm for measuring bending angles. One end of the strips was attached to a vertical glass plate to generate a cantilever structure. Upon NIR irradiation, the bending angle was calculated according to the schematic illustration in Figure S7, Supporting Information.

### Photocatalytic H_2_ Evolution

The E_2.5_C_10_ and the E_0_C_10_ hydrogels were used to simulate flower stems, respectively. The bionic sunflower was placed in a quartz reactor containing an aqueous solution of triethanolamine (TEOA, Sinopharm Group). The device was illuminated using a broadband 150 W Newport xenon lamp (CEL‐HXUV300) with wavelengths ranging from 300–1100 nm. The quantity of H_2_ generated at each interval was measured by gas chromatography (GC) (Shimadzu GC‐2014). Wavelength‐dependent H_2_ evolution and AQY were measured at the designated monochromic light by using appropriate bandpass filters (400, 420, 450, and 500 nm, respectively). The AQY was calculated by using the following equation:

(2) $$AQY=\frac{2\times \mathrm{number}\;\;\mathrm{of}\;\;\mathrm{evolved}\;\;H_{2}}{\mathrm{number}\;\;\mathrm{of}\;\;\mathrm{incident}\;\;\mathrm{photons}}\times 100\%$$

## Conflict of Interest

The authors declare no conflict of interest.

## Supporting information

Supporting Information

Supporting Movie 1

Supporting Movie 2

Supporting Movie 3

Supporting Movie 4

Supporting Movie 5

## Data Availability

The data that support the findings of this study are available in the Supporting Information material of this article.
