# Supplementary material for: Light‐Fueled Hydrogel Actuators with Controlled Deformation and Photocatalytic Activity
Source: Adv Sci (Weinh). 2022 Oct 17;9(34):2204730. doi: 10.1002/advs.202204730 (PMC9731706; doi:10.1002/advs.202204730)
Supplement: Supplementary file 1 — Supporting Information [file ADVS-9-2204730-s004.pdf]

ADVANCED SCIENCE
Open Access

Supporting Information

for *Adv. Sci.*, DOI 10.1002/advs.202204730

Light-Fueled Hydrogel Actuators with Controlled Deformation and Photocatalytic Activity

Pengyu Chen, Qiushi Ruan, Rasool Nasseri, Hanning Zhang, Xufeng Xi, Huan Xia, Gang Xu, Qian Xie, Chengjie Yi, ZhengMing Sun, Hamed Shahsavan* and Wei Zhang**

Supporting Information

Light-fueled Hydrogel Actuators with Controlled Deformation and Photocatalytic Activity

Pengyu Chen,‡ Qiushi Ruan,‡ Rasool Nasseri, Hanning Zhang, Xufeng Xi, Qian Xie, Chengjie Yi, ZhengMing Sun, Hamed Shahsavan,* Wei Zhang**

P. Y. Chen, Prof. Q. S. Ruan, H. N. Zhang, X. F. Xi, Q. Xie, C. J. Yi, Prof. Z. M. Sun, Prof. W. Zhang
Jiangsu Key Laboratory of Advanced Metallic Materials, School of Materials Science and Engineering, Southeast University, Nanjing, 211189, PR China
E-mail: zmsun@seu.edu.cn; w69zhang@seu.edu.cn
R. Nasseri, Prof. H. Shahsavan
Department of Chemical Engineering, and Waterloo Institute for Nanotechnology, University of Waterloo, Waterloo, Ontario N2L 3G1, Canada;
E-mail: hshahsav@uwaterloo.ca

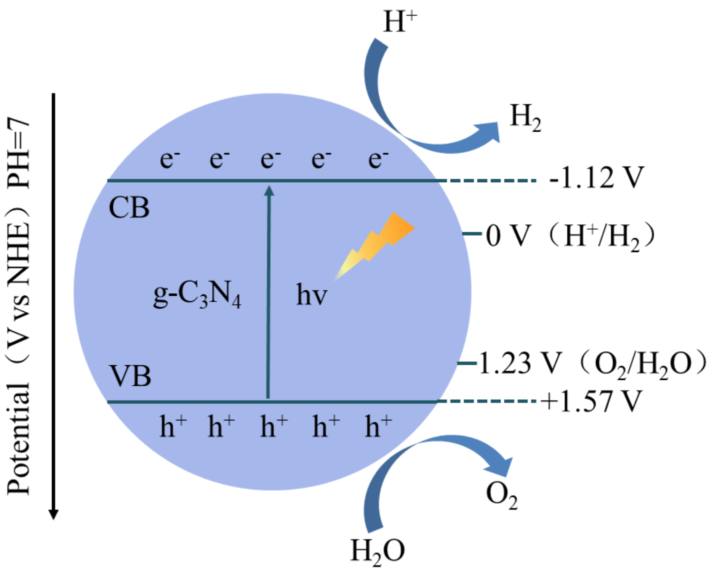

Figure S1. Schematic diagram of g-C_3N_4 photocatalytic hydrogen generation and energy band.

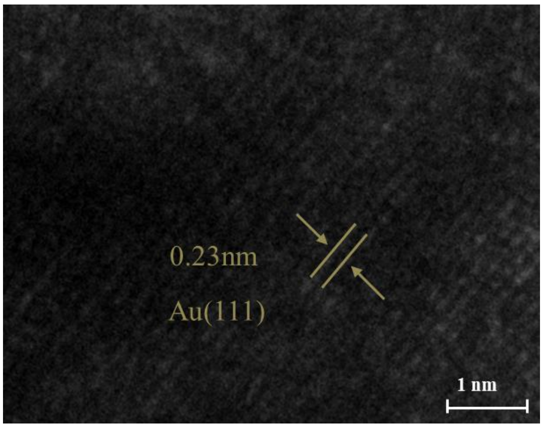

Figure S2. Magnified TEM image of the Au NPs.

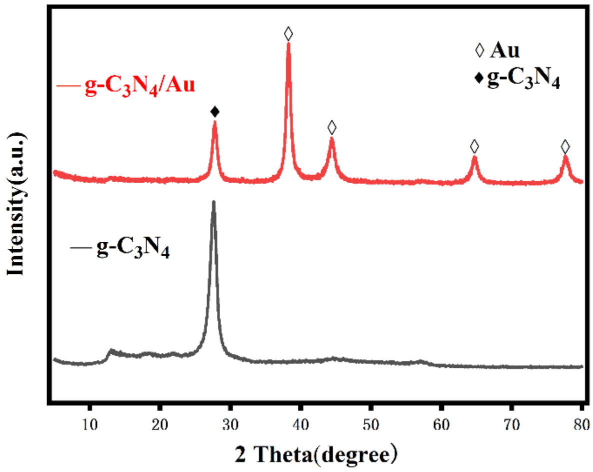

Figure S3. XRD patterns of Au/g-C_3N_4 and pure g-C_3N_4.

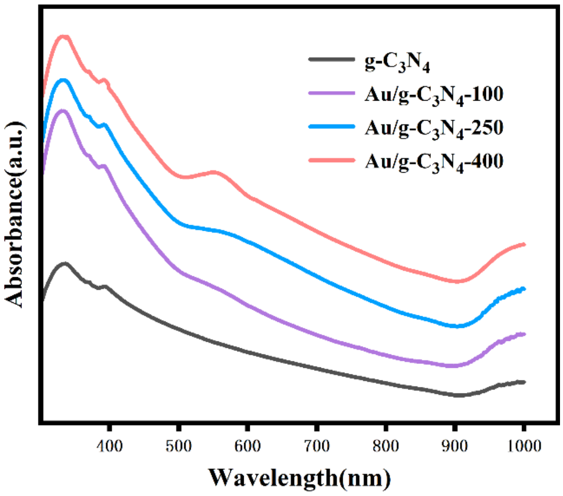

Figure S4. UV-vis spectra of pure g-C_3N_4 and Au/g-C_3N_4 synthesized with different contents of chloroauric acid (100, 250, 400 μL) in precursor solutions dispersions in deionized water.

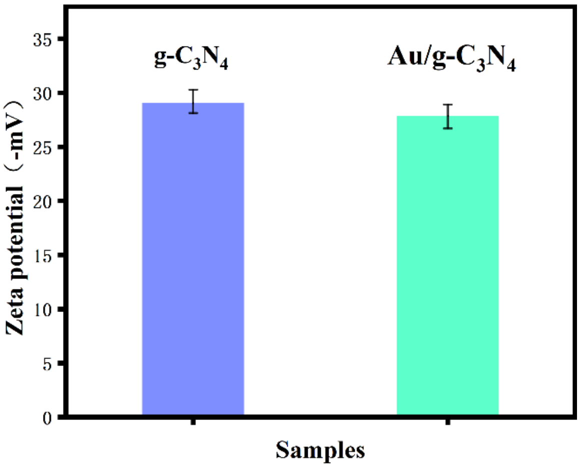

Figure S5. The Zeta potentials of g-C_3N_4 and Au/g-C_3N_4 dispersions in deionized water.

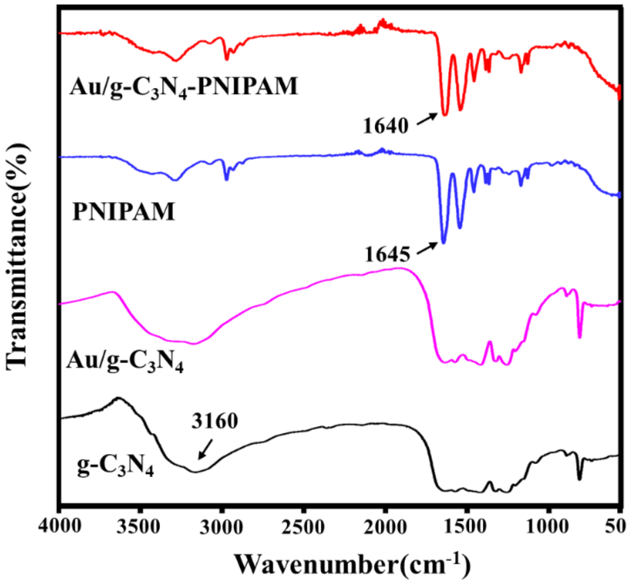

Figure S6. The FT-IR spectra of g-C_3N_4, Au/g-C_3N_4, PNIPAM, and the Au/g-C_3N_4-PNIPAM hydrogel.

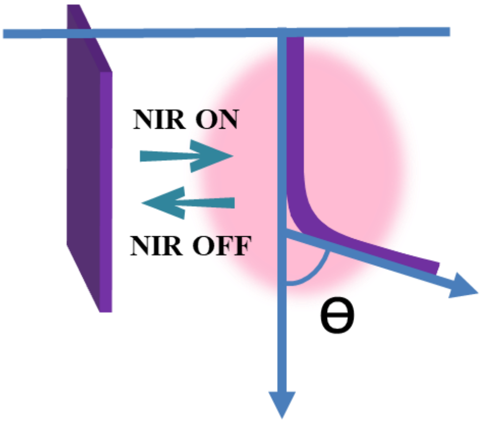

Figure S7. Schematic illustration of the gradient hydrogels bending angle (θ) under NIR light irradiation.

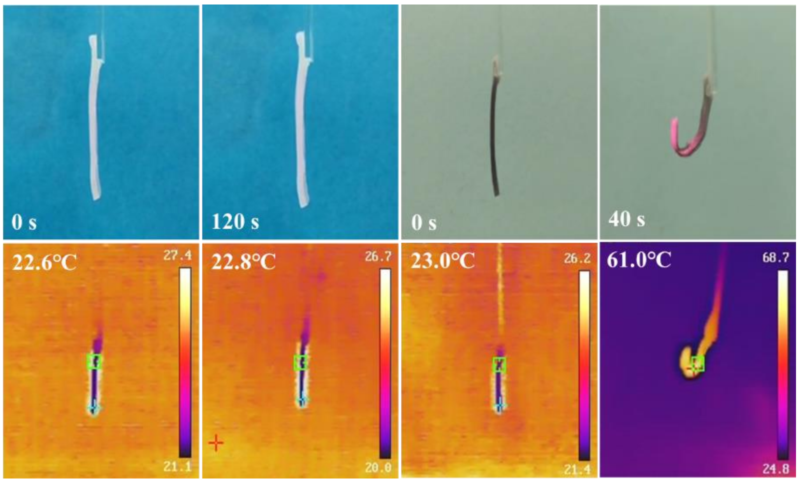

Figure S8. Real and infrared images of the $E_{2.5}C_{10}$ hydrogel actuator and the control sample undoped with Au NPs upon exposure to 808 nm NIR light (0.5 W cm^{-1}).

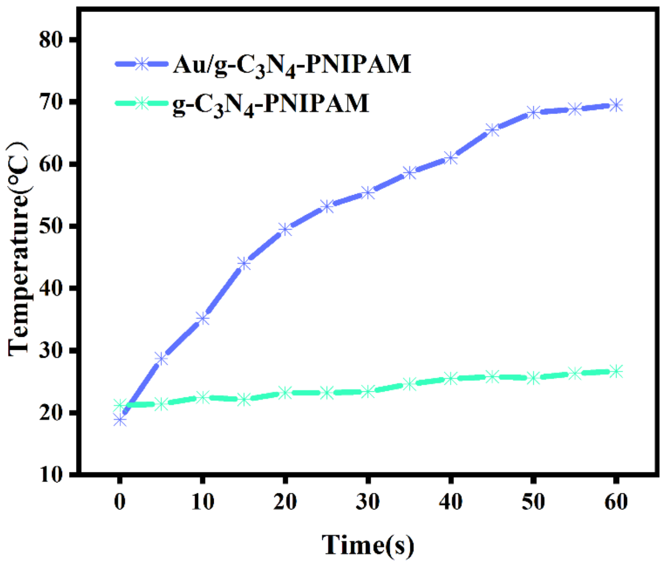

Figure S9. Temperature variation with an exposure time of the $E_{2.5}C_{10}$ hydrogel and g-C_3N_4-PNIPAM hydrogel under 808 nm NIR irradiation with 0.5 W cm^{-2}.

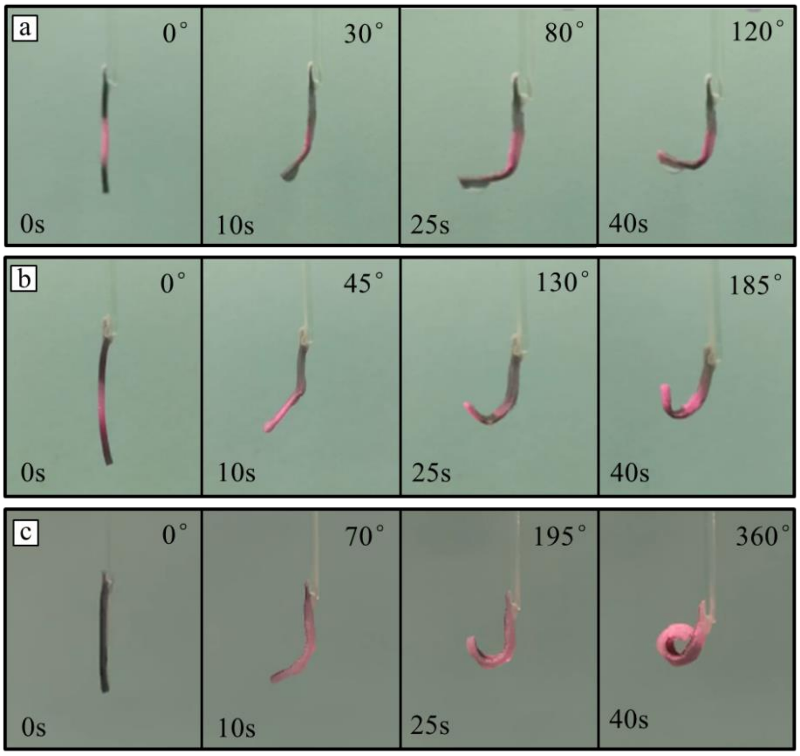

Figure S10. Actuation of gradient hydrogel actuators prepared at different voltages: (a) the E_2C_{10}, (b) the $E_{2.5}C_{10}$, and (c) the E_3C_{10} upon exposure to the 808 nm NIR light with 0.5 W cm^{-2}.

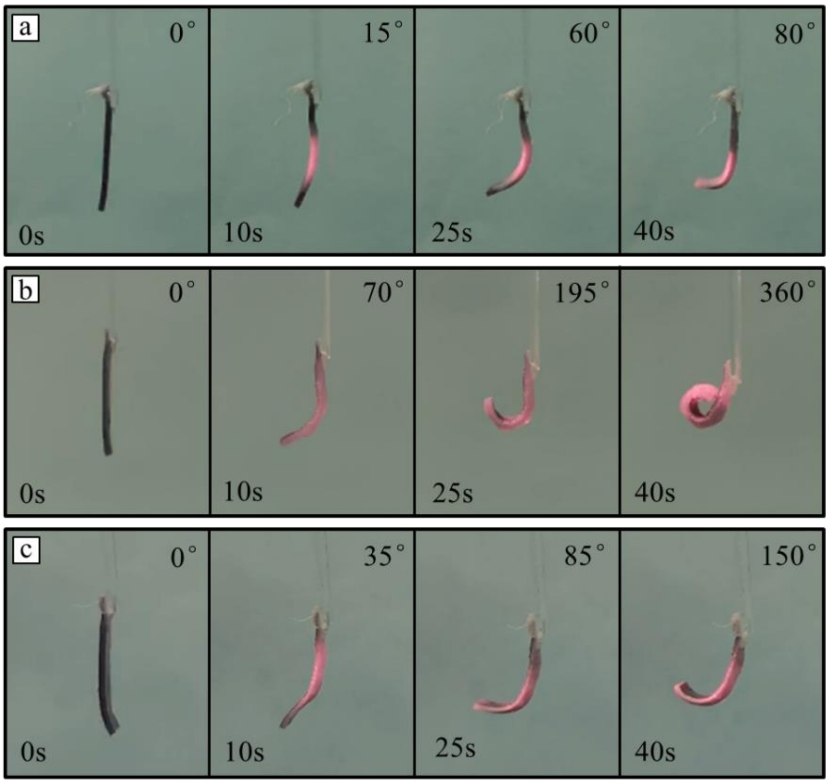

Figure S11. Actuation of gradient hydrogel actuators prepared with different Au/g-C_3N_4 concentrations: (a) the E_3C_5 (b) the E_3C_{10}, and (c) the E_3C_{15} upon exposure to the 808 nm NIR light with 0.5 W cm^{-2}.

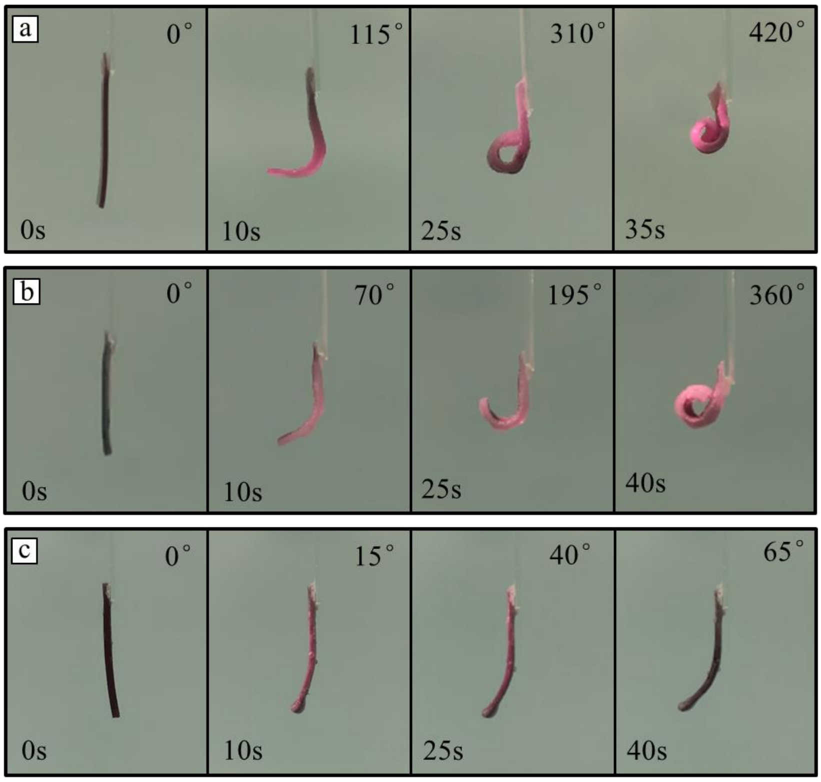

Figure S12. Actuation of gradient hydrogel actuators prepared with different

thicknesses: (a) 0.7, (b) 1.0, and (c) 1.4 mm upon exposure to the 808 nm NIR light with 0.5 W cm^{-2}.

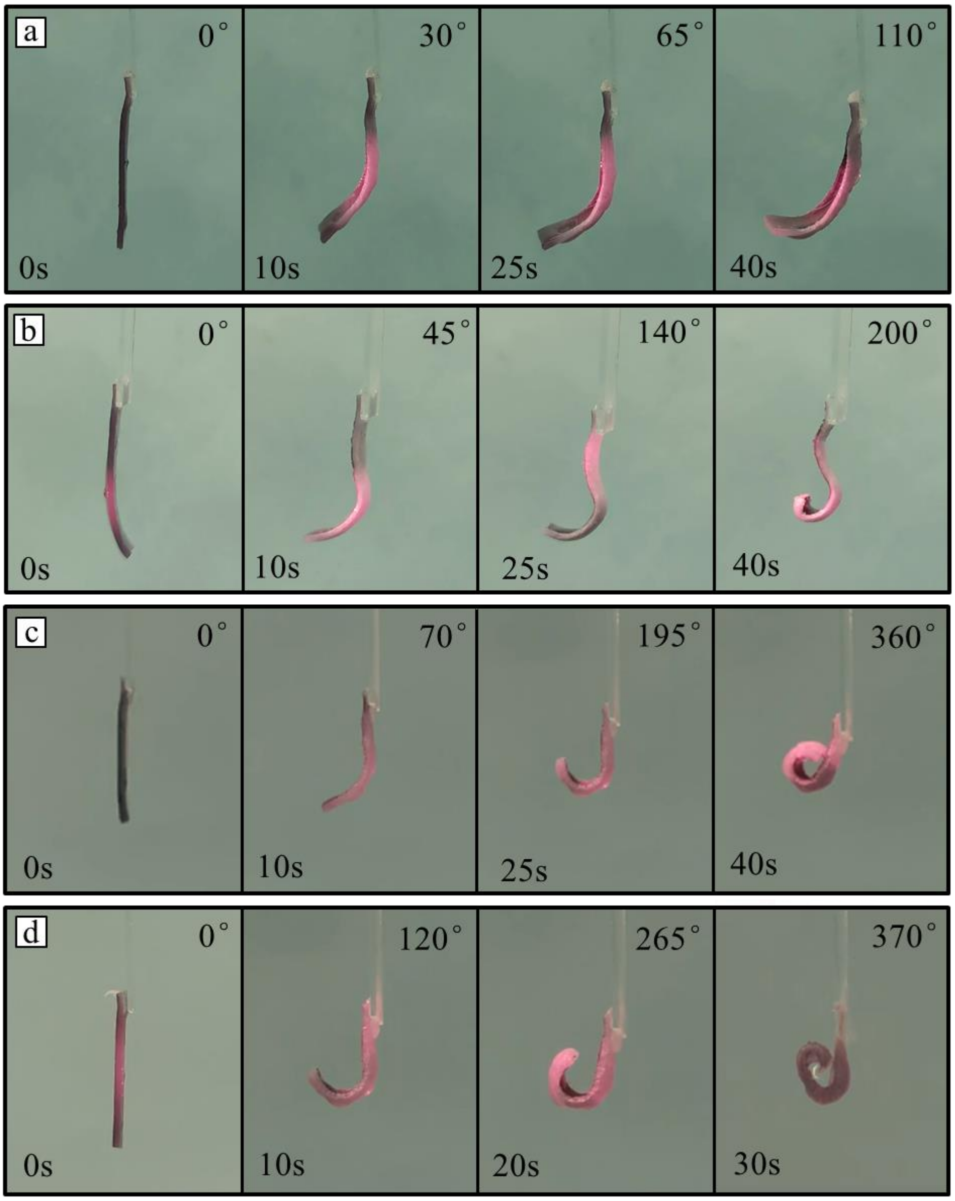

Figure S13. Actuation of the E_3C_{10} hydrogel actuators exposed to different power density: (a) 0.1, (b) 0.2, (c) 0.5 and (d) 0.8 W cm^{-2}.

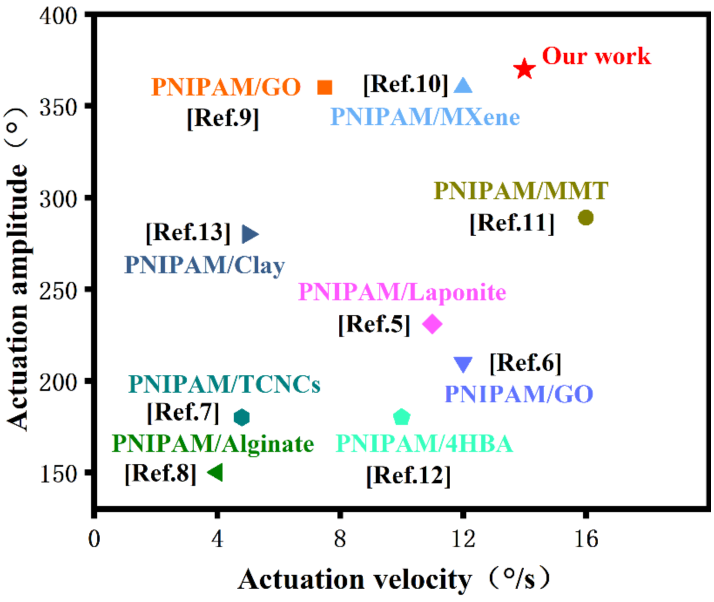

Figure S14. Comparison of actuation velocity and actuation amplitude of different hydrogel actuators. The detailed information is shown in Table S2.

Table S1. Comparison of g-C_3N_4-based 3D structures photocatalytic AQY performance

**Sample**	**AQY(%)**	**Wavelength(nm)**	**Lamp power(W)**	**Ref(SI)**
PNIPAM/g-C_3N_4	1.88	420	300W	1
3D porous C_3N_4	5.1	420	300W	2
PDMA/g-C_3N_4	1.0	405	50W	3
Porous fiber-like g-C_3N_4	2.42	420	500W	4
This work	5.39	420	150W	

Table S2. Comparison of actuation performance of different hydrogel actuators

**Hydrogel**	**Actuation amplitude (°)**	**Actuation velocity (°/s)**	**Ref**
PNIPAM-Laponite	231	11	5
PNIPAM-GO	210	12	6
PNIPAM-TCNCs	180	4.8	7
PNIPAM-Aliginate	150	4	8

PNIPAM-GO	360	7.5	9
PNIPAM-MXene	360	12	10
PNIPAM-MMT	289	16	11
PNIPAM-4HBA	180	10	12
PNIPAM-Clay	280	5	13
PNIPAM-Au/g-C_3N_4	370	13	This work

Supporting Movies:

Movie S1: Light-fueled flower-shaped hydrogel actuator in water under 808 nm NIR with 0.8 W cm^{-2} intensity.

Movie S2: Light-fueled soft end effector that grips and lifts a cargo in water when exposed to 808 nm NIR with 0.8 W cm^{-2} intensity.

Movie S3: Light-fueled hydrogel paddles enable the motion of a boat-like structure on the water under 808 nm NIR irradiation with 0.8 W cm^{-2} intensity.

Movie S4: Light-driven hydrogel walker on ratchet surface in water under 808 nm NIR irradiation with 0.8 W cm^{-2}.

Movie S5: The flower-shaped construct bends when exposed to the light source to maximize energy harvesting underwater.

Reference

[1] L. Lei, W. J. Wang, C. Wang, H. Q. Fan, A. K. Yadav, N. Hu, Q. Zhong and P. Muller-Buschbaum, *J. Mater. Chem. A*, 2020, **8**, 23812-23819.

[2] J. Xu, L. W. Zhang, R. Shi and Y. F. Zhu, *J. Mater. Chem. A*, 2013, **1**, 14766-14772.

[3] Q. Cao, J. Barrio, M. Antonietti, B. Kumru, M. Shalom and B. Schmidt, *ACS Appl. Polym. Mater*, 2020, **2**, 3346-3354.

[4] H. L. Dou, D. Long, S. H. Zheng and Y. P. Zhang, *Catal. Sci. Technol*, 2018, **8**, 3599-3609.

[5] Y. Tan, D. Wang, H. X. Xu, Y. Yang, W. L. An, L. N. Yu, Z. X. Xiao and S. M. Xu, *Macromol. Rapid Commun*, 2018, **39**, 6.

[6] X. M. He, Y. Sun, J. H. Wu, Y. Wang, F. Chen, P. Fan, M. Q. Zhong, S. W. Xiao, D. Zhang, J. T. Yang and J. Zheng, *J. Mater. Chem. C*, 2019, **7**, 4970-4980.

[7] K. W. Mo, M. He, X. D. Cao and C. Y. Chang, *J. Mater. Chem. C*, 2020, **8**, 2756-2763.

[8] W. J. Zheng, N. An, J. H. Yang, J. X. Zhou and Y. M. Chen, *ACS Appl. Mater. Interfaces*, 2015, **7**, 1758-1764.

[9] Y. Yang, Y. Tan, X. Wang, W. An, S. Xu, W. Liao and Y. Wang, *ACS Appl. Mater. Interfaces*, 2018, **10**, 7688-7692.

[10] P. Xue, H. K. Bisoyi, Y. H. Chen, H. Zeng, J. J. Yang, X. Yang, P. F. Lv, X. M. Zhang, A. Priimagi, L. Wang, X. H. Xu and Q. Li, *Angew. Chem.Int. Edit*, 2021, **60**, 3390-3396.

[11] J. Liu, W. Xu, Z. Kuang, P. Dong, Y. Yao, H. Wu, A. Liu and F. Ye, *J. Mater. Chem. C*, 2020, **8**, 12092-12099.

[12] R. C. Luo, J. Wu, N. D. Dinh and C. H. Chen, *Adv. Funct. Mater*, 2015, **25**, 7272-7279.

[13] H. Zhao, Y. M. Huang, F. T. Lv, L. B. Liu, Q. Gu and S. Wang, *Adv. Funct. Mater*, 2021, 31, 2105544.
